# Nest Enlargement in Leaf-Cutting Ants: Relocated Brood and Fungus Trigger the Excavation of New Chambers

**DOI:** 10.1371/journal.pone.0097872

**Published:** 2014-05-15

**Authors:** Daniela Römer, Flavio Roces

**Affiliations:** Department of Behavioural Physiology and Sociobiology (Zoology II), Biocenter, University of Würzburg, Würzburg, Germany; University of Arizona, United States of America

## Abstract

During colony growth, leaf-cutting ants enlarge their nests by excavating tunnels and chambers housing their fungus gardens and brood. Workers are expected to excavate new nest chambers at locations across the soil profile that offer suitable environmental conditions for brood and fungus rearing. It is an open question whether new chambers are excavated in advance, or will emerge around brood or fungus initially relocated to a suitable site in a previously-excavated tunnel. In the laboratory, we investigated the mechanisms underlying the excavation of new nest chambers in the leaf-cutting ant *Acromyrmex lundi*. Specifically, we asked whether workers relocate brood and fungus to suitable nest locations, and to what extent the relocated items trigger the excavation of a nest chamber and influence its shape. When brood and fungus were exposed to unfavorable environmental conditions, either low temperatures or low humidity, both were relocated, but ants clearly preferred to relocate the brood first. Workers relocated fungus to places containing brood, demonstrating that subsequent fungus relocation spatially follows the brood deposition. In addition, more ants aggregated at sites containing brood. When presented with a choice between two otherwise identical digging sites, but one containing brood, ants' excavation activity was higher at this site, and the shape of the excavated cavity was more rounded and chamber-like. The presence of fungus also led to the excavation of rounder shapes, with higher excavation activity at the site that also contained brood. We argue that during colony growth, workers preferentially relocate brood to suitable locations along a tunnel, and that relocated brood spatially guides fungus relocation and leads to increased digging activity around them. We suggest that nest chambers are not excavated in advance, but emerge through a self-organized process resulting from the aggregation of workers and their density-dependent digging behavior around the relocated brood and fungus.

## Introduction

Leaf-cutting ants build the most complex underground nests among ants. Their nests may consist of up to eight thousand underground chambers housing their symbiotic fungus, brood embedded within the fungus and in several species, also the colony's refuse [1]–[3]. Huge nests with millions of individuals and thousands of fungus chambers are generally excavated by colonies of the genus *Atta* [4], [5], while colonies of the genus *Acromyrmex* excavate smaller nests composed of one or up to tens of chambers [6], [7] with mature colony sizes between a few thousand [8] and one to two hundred thousand individuals [9], [10]. All these nests are composed of two kinds of structures: oblong, narrow tunnels and spherical chambers with a flat bottom and a dome shaped ceiling, but each species has its own specific nest architecture by which it can be identified [4], [5], [7], [11]–[14]. *Atta* nests consist of a net of main tunnels leading downwards to deeper soil regions. Nest depths of 8 m have been reported for *Atta laevigata* [4], [14]. These main tunnels connect to the nest chambers, which are oriented laterally to tunnels, mostly by one short and narrow branched off tunnel called peduncle, which end in the lower part of the chamber [4], [14], [15]. The main tunnels can have blind endings and a recent study using cement casts from *Atta laevigata* and *Atta capiguara* nests showed that these tunnels may have the beginnings of branched off peduncles, which end blind without excavated chambers [14]. There are also tunnels that lead farther downwards than the fungus chamber zone and are thought to reach the water table [2], [15], [16], as well as horizontal foraging tunnels of considerable length [4], [14], [16]. *Acromyrmex* nests are generally shallower, with fungus chambers found close to the soil surface (5–50 cm) [11], [13], [17], [18], but nest depths also reaching 2–5 m have been reported for some species [7], [19], [20]. The nest tunnel system, while not as complex as that of *Atta* nests, also extends beyond the existing garden zone and some tunnels end blind.

Whether mature nests consist of thousands (*Atta*) or just a few (*Acromyrmex*) chambers, the founding nest is a single, downward leading tunnel of 10–30 cm in length connected to a small chamber, which is excavated by a new queen after her mating with several males [21], [22]. Mating flights take place in spring during the hot months and after heavy rains [12], [23], when both the temperature and humidity of the soil are high and conditions are well suited to successfully rear fungus gardens and brood. Information on how nests are enlarged after this first step is scarce though more is known for *Atta* than for *Acromyrmex* species. The process of nest enlargement in ants is not centrally coordinated and appears to be self-organized with workers reacting to local stimuli without knowledge of the complete structure [24]–[26]. When the first leaf-cutting ant workers appear 8–12 weeks after colony founding [27], [28], they are responsible for further nest enlargement, achieved by the excavation of tunnels, mostly leading downwards, and the excavation of new fungus chambers at deeper soil layers [27], [29]. *Acromyrmex* species are thought to enlarge their nests by building a few interconnected fungus chambers close to the surface. For example *A. lundi*, which has relatively simple mature nests with a large (diameter 50 cm) central chamber linked by tunnels to a few satellite chambers, had only created a small central chamber with tunnels originating from it, but no satellite chambers, within 1–2 years after colony foundation [11].

Ants may increase the size of their nests in two ways, either enlarging existing chambers or excavating new ones. Mature fungus chambers in *Atta* species usually have a diameter of ∼30 cm while chambers in more superficially nesting *Acromyrmex* species may reach a diameter of ∼50 cm. The extent of chamber enlargement seems to have an upper limit. For example in a field nest of *Atta*, where chamber density was observed to be high, sometimes neighboring chambers were only separated by a very thin layer of soil [14], [30]. Fusing the chambers together could have been achieved by the ants at a low energetic cost, yet this barrier was not breached. It remains to be discovered what the limiting factors are for enlarging an existing chamber. Cassil [31] for example proposed that smaller chamber sizes benefit colony communication in the fire ant, *Solenopsis invicta*. Large fungus chambers may have a reduced supply of fresh air, because of the diffusive movement of respiratory gases that need to reach the center of the fungus garden [14], [32]. As a result, at least at one time and likely at many intervals in the development of these colonies, their growth trajectory will exceed the space available within a single chamber and a new one must be constructed.

It is an open question whether new nest chambers are excavated in advance as colonies grow, or whether they emerge around an incipient cache of brood and/or fungus. In addition to the stimulus resulting from insufficient space there are three other non-mutually exclusive scenarios for the relocation of brood and fungus from an existing chamber, and the potential excavation of a new chamber around them. First, pathogens may infect a fungus garden, and workers may remove and relocate healthy fungus pieces and brood. Second, the microclimatic conditions inside the fungus chamber may become unsuitable for brood and fungal development. Third, even when the conditions are not unsuitable, workers may find, or search for, more favorable conditions at a different location. All these four scenarios would potentially lead to brood and fungal deposition at a new site in the nest, i.e. in an existing tunnel, and to the subsequent excavation around them to create a chamber. However, empty chambers have been reported in field nests of a number of leaf-cutting ant species [1], [2], [4], [5], [7], [20], [33], [34]. One possibility is that such chambers were constructed around relocated items, and later emptied because of changing environmental conditions [20], presence of pathogens or fungus decay. Alternatively, ants might start the excavation of a chamber in advance upon finding a suitable place for their fungus and brood, as a direct reaction to local abiotic stimuli such as temperature or humidity. Ideal conditions for in vitro fungus rearing are temperatures between 20 and 30°C [35], [36] and in fact, leaf-cutting ants choose places with temperatures between 21 and 25°C when they relocate fungus and brood [37]. They also prefer relative humidities close to saturation for fungus rearing [38]. Given a choice between alternative sites, leaf-cutting ants prefer to dig at temperatures between 20–30°C, which may lead to a concentration of digging activity in soil layers of the preferred temperature range [39]. The nest enlargement in many *Atta* and some *Acromyrmex* species also takes place at deeper soil layers, which have a higher moisture content [27], [29]. More superficially nesting *Acromyrmex* species might conserve moisture in the soil surrounding their nests by accumulating leaf-litter on the nest surface, by plugging nest entrances, or by modifying the structure of the nest mound [13], [40]–[42]. To ensure proper conditions for brood and fungus rearing, and with it the survival of the colony, leaf-cutting ants even track their preferred temperature and humidity values across an existing nest and brood and fungus are relocated accordingly [16], [20], [37], [43].

The question arises whether abiotic environmental stimuli alone are sufficient to trigger digging of a new nest chamber in advance at a suitable location. Under controlled laboratory conditions, workers of *Acromyrmex lundi* with neither brood nor fungus excavated only tunnels, but not chambers [44], [45]. Chambers were excavated as soon as the ants were allowed to relocate symbiotic fungus inside a digging arena, and digging activity concentrated around the deposited fungus. This suggests that beyond abiotic stimuli, contents to be stored are needed for the emergence of a nest chamber. We hypothesize that a suitable microclimate at a potential chamber location is not sufficient to trigger the excavation of a chamber, but that the contents to be stored, brood or fungus, are needed at this location to initiate chamber excavation. We propose that if chamber content is relocated to an already existing tunnel, excavation to generate further space should follow. To investigate this two-step process (relocation followed by excavation), we designed a series of experiments that first investigate the relocation of brood and fungus and then quantify the digging activity and chamber formation triggered by the relocated items.

The separate analysis of the relocation and excavation processes was necessary because it was unknown whether brood and fungus would be relocated simultaneously or sequentially, either of which might have distinct influences on subsequent digging behavior. Relocation comprises the removal of items at one place and their deposition at another. We first investigated the removal of items by exposing brood and fungus to unsuitable environmental conditions, using low temperature in a first experiment, and low air humidity in a second. We found that the ants exhibited a strong preference to remove the brood first. Because brood and fungus are maintained together in natural nests (as the young brood need to feed on the fungus), we expected that the subsequently removed fungus would be deposited near the relocated brood. We evaluated fungus deposition in binary-choice experiments offering two sites with suitable environmental conditions, only one containing brood. Two last experimental series were designed to evaluate whether chamber content triggers chamber excavation by quantifying the digging activity and shapes of excavated structures at two suitable sites offered in binary-choice experiments. One site contained brood, while the other did not, both in the presence or absence of fungus. Based on our findings we propose a density-dependent mechanism for the emergence of nest chambers through a self-organized process, with relocated brood and fungus acting as cues that elicit worker aggregation at their deposition sites, indirectly influencing the intensity of digging activity.

## Materials and Methods

Experiments were performed in the laboratory between June 2010 and December 2011 with leaf-cutting ants of the species *Acromyrmex lundi*. This species is not protected under the Convention on International Trade in Endangered Species of Wild Fauna and Flora (CITES). Colonies were collected in Argentina in 2007 on privately owned land with the owner giving permission for their collection. They were reared at the Biocenter of the University of Würzburg, Germany in a walk-in environmental chamber at 25°C, 50% air humidity and a 12L∶12D cycle. To control for possible effects of body size on behavioral performance, only medium sized workers (mean body mass calculated from a ubiquitous sample of medium sized workers taken from the colonies for weighing: 5.3 mg±1.2 mg SD, n = 80) were used in the experiments described below. All experiments were performed with worker groups from large laboratory colonies. We realize that colonies of this species build relatively superficial nests with a few and sometimes just one nest chamber. However, we argue that colonies from this species, probably as well as from all leaf-cutting ant species, are confronted during their ontogeny with the need to enlarge their nests either by increasing the size of an existing chamber, or by excavating a new one, or both. Also, previous related studies from our lab were conducted on *A. lundi* [44], [45], so that direct comparisons are possible.

After each assay the worker groups were not reintroduced into the colonies, so that each assay was considered independent from each other. To control for possible colony differences, 3 colonies were used and, if not otherwise stated, worker groups from all 3 colonies were used for each experiment. We also tested for possible colony effects, and the results of these tests are included in the appropriate figure captions. Since no colony effects were found, data from all colonies was pooled for statistical analysis.

### (a) Determination of relocation preference for brood or fungus

As previously indicated, the sequence of brood and fungus relocation in natural nests is unknown. Since individual workers necessarily relocate single brood items and pieces of fungus separately, it is an open question whether workers prefer to relocate brood or fungus first when presented with a choice. The kind of items relocated first may distinctly influence the subsequent digging behavior at the deposition site. To evaluate the worker's preferences during relocation we first evaluated, in two independent experimental series, whether ants prioritize one item over the other during relocation to suitable nest conditions or if they were relocated simultaneously. If the former is the case, the preferentially removed item would be present at a new site first, i.e., within a nest tunnel, and might initially trigger the subsequent chamber excavation. In both series, removal was induced by exposing brood and fungus to unsuitable conditions, either low temperature or low air humidity, and their relocation quantified. Based on the outcome of these experiments we chose the item to be used as trigger for the excavation of a chamber in the digging experiments.

#### Temperature induced relocation experiment

**Figure 1:**
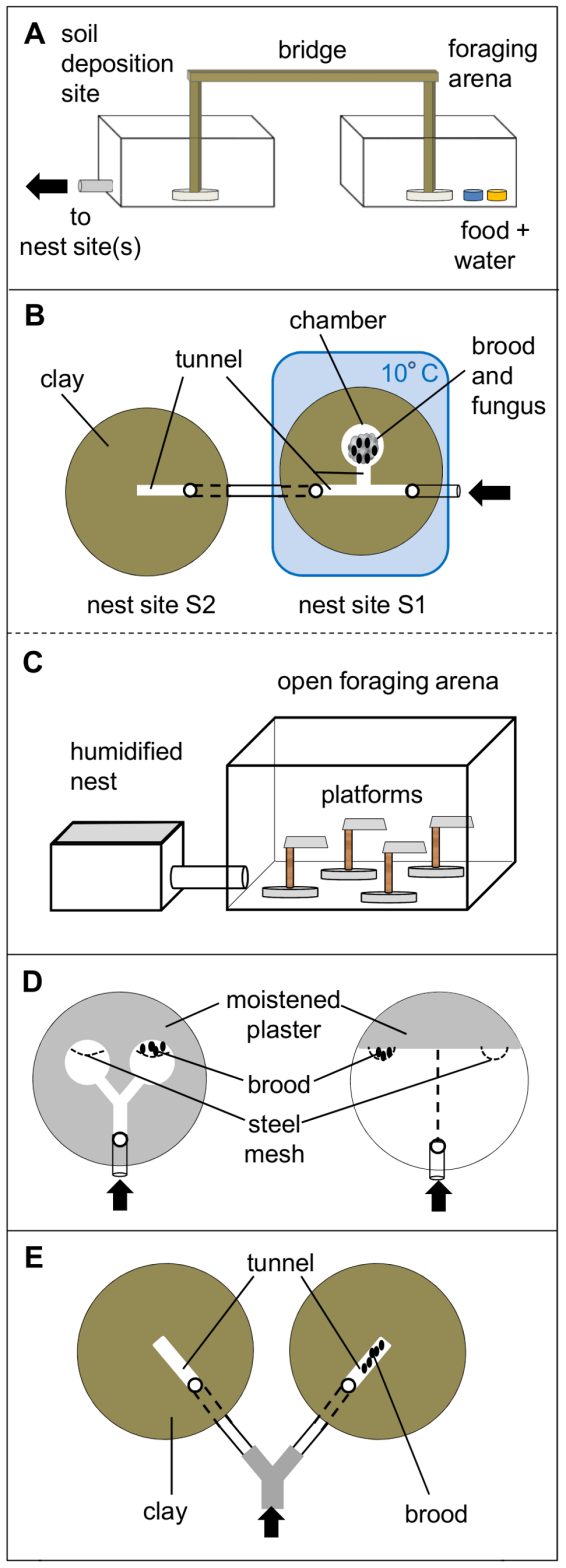
Experimental setups (arrows indicate the direction of entering ants). (a) Foraging area consisting of a soil deposition site (left) and a foraging arena (right). Here the arrow indicates the entry to experimental arena(s). (b) Nest sites for the temperature induced relocation experiment (Experiment 1): left – nest site with cut-out tunnel, at room temperature; right – nest site with cut out tunnels and chamber containing fungus and brood, placed on a cooling plate. (c) Setup for humidity induced relocation experiment (Experiment 2): left – humidified nest with moistened pebbles; right – open foraging arena with 4 experimental platforms. Ants were placed into the foraging arena at the beginning of the experiment. (d) Plaster nest sites for fungus relocation experiment: left – nest site with 2 small chambers, one containing brood (Experiment 3); right – nest site with 1 big chamber, one side containing brood (Experiment 4). (e) Clay nest sites for digging experiments, only one tunnel containing brood (Experiments 5 and 6).

In the first series, the dynamics of fungus and brood transport were quantified when both were simultaneously exposed to a low temperature to initiate relocation behavior (Experiment 1, Fig. 1a and 1b). The experimental setup was as follows. To simulate a small nest, two round plastic arenas (diameter 15 cm, height 1 cm, henceforth called nest site 1 (S1) and nest site 2 (S2)) were filled with moist clay (Claytec Baulehm gemahlen 0–0.5 mm, Viersen, Germany, water content 18%, air humidity in nest site 99.9%) and connected to each other with a piece of tubing (length 10 cm). Two separate nest sites were necessary because site S1 was exposed to a lower temperature during the experiment, while site S2 was maintained at room temperature to offer a suitable microclimate. In nest site S1, we artificially constructed a main tunnel (7×1×1 cm), a short side tunnel (1×1×1 cm) and a chamber (diameter 5 cm) by cutting these spaces out of the clay (Fig. 1b). In nest site S2 only a tunnel (4×1×1 cm) was cut out. Prior to the experiment 0.5 g of fungus (without brood and gardening workers) and 20 pupae, both freshly removed from one of the large colonies, were placed inside the chamber in nest site S1. This site was then connected to a foraging area that consisted of two boxes (19×19×9 cm), linked by a wooden bridge. The first box contained an ample supply of water as well as honey water and will therefore be called ‘foraging arena’. When restricted to only one foraging box, workers tend to spoil their food supply by mixing it with excavated clay pellets, which would negatively influence the workers' survival rate during the experiments. To prevent this situation a second box was added for soil deposition (Fig. 1a).

At the beginning of each assay 100 workers, collected from one of the three colonies, were released in the foraging arena. They could move freely across the wooden bridge into the soil deposition site and from there into and out of the nest. After two hours of familiarization time, the temperature in site S1, which had been placed on a cooling plate connected to a water bath, was lowered from room temperature (ca. 20°C) to 10°C. This temperature was chosen to induce relocation because previous work indicated that workers of the related species *Acromyrmex heyeri* avoid this temperature and relocate brood or fungus to warmer places [37]. Fifteen replicates were performed, using 5 worker groups from each colony. After 22 hours, the amount of relocated fungus into nest site S2 was weighed to the nearest 0.1 mg, and the number of relocated brood were counted. These were then converted to proportion (%) of the total content placed in nest site S1.

#### Humidity induced relocation experiment: individual choices

It is important to note that the fungus in chambers of natural nests is a dense connected mass of hyphae, and that ants need to cut a transportable piece from the large mass for relocation. Brood might therefore be relocated first not necessarily because of a preference, but simply because they are just easier to pick-up and remove. To control for this effect, we performed the second experimental series using small, transportable pieces of fungus and observed the removal decision in real time. As low air humidity was observed to initiate a quick removal of items in preliminary experiments, it was used instead of low temperature as an unsuitable environmental factor to trigger removal. Because the experiment was easier to implement outside of the nest, one pupa and one piece of fungus were simultaneously exposed in a foraging arena (Experiment 2, Fig. 1c). Removal preferences of single workers were quantified in individual choice experiments. A plastic box with a lid (9×9×6 cm) acted as a nest site, with its bottom filled with moistened pebbles to offer humid conditions (air humidity levels in the nest site, close to saturation, 99.9%). It was connected to a foraging arena (an open box 19×19×9 cm) with humidity levels corresponding to room conditions (∼50%), at which fungus and brood faced the threat of desiccation. Four platforms, each consisting of a plastic square (1.5×1.5 cm) glued on top of a 4.5 cm high wooden stick were placed in the foraging arena. In each assay 50 workers were released there and could explore it as well as the nest site for 1 h. Then, a piece of fungus and a brood item were placed on a randomly chosen platform. The mass of a brood item was 7.2±0.18 mg (mean ± SE), and that of a fungus piece 13.9±0.33 mg (mean ± SE). An ant, upon walking up the wooden stick to the platform, would encounter both items simultaneously. It was then noted which item was picked up first (and relocated to the nest site), and the time it took for the second item to be picked up by a different worker. Workers that relocated items were carefully removed with forceps after depositing their load in the nest. Tests were performed over 1 h, with the platforms chosen at random each time. In total, 6 assays were performed using 3 different colonies (2 assays per colony) and a total of 101 pupa/piece of fungus pairs (pairs per colony: 41, 30, 30) were tested. Both experiments (1 and 2), although using different stimuli to trigger the ants' responses, were designed to evaluate the removal preference and not the final deposition of the items.

### (b) Brood as a cue for fungus relocation

The deposition of items during a relocation process was evaluated in the next two experiments. Because workers showed a preference for brood relocation in the previous experiments, which could lead to the presence of brood at an alternative site first, only the influence of deposited brood on the subsequent fungus relocation was investigated. The brood in a fungus chamber is usually embedded into the fungal mass [40], [46], with workers planting hyphae on the larval body [47]. As a consequence, we would expect that workers transport fungus pieces to a site where brood had been previously relocated to. Brood may therefore act as an orientation cue for workers relocating fungus. The subsequent accumulated fungal volume around the brood should then have an influence on the excavation of space at this site. Therefore, it was important to first demonstrate that relocation of fungus, as we expected, will follow relocation of brood. A set of two experimental series was performed without the involvement of digging activity. In the first experiment, ants were induced to relocate fungus from unsuitable conditions (low air humidity) and had the choice between two nest sites, one containing brood and the other without brood (Experiment 3, Fig. 1d, left), both offering suitable environmental conditions (temperature ∼25°C, air humidity close to saturation). The rationale of offering two sites instead of one was to mimic more natural conditions, since natural nests may offer more than one site for a potential relocation.

The nest site consisted of a round plastic arena (diameter 15 cm, height 1 cm) filled with plaster (Sakret Bau- und Hobbygips, Berlin, Germany). We chose this material to prevent the ants from digging. A Y-shaped tunnel with a nest chamber (diameter 5 cm) at each end was cut out of the material, and pieces of steel mesh were fastened into the plaster to separate a part of each chamber (Fig. 1d, left). The plaster was remoistened with 10 ml of demineralized water (resulting air humidity levels 99.9%) and 20 pupae were placed behind the mesh in one of the nest chambers, so as to prevent their removal when workers entered the chamber during the assays. The nest site was then connected to a foraging arena (an open plastic box, 19×19×9 cm) containing an ample supply of water and honey water. At the beginning of each assay, a group of ants consisting of 50 medium and 10 minima workers (mean size 0.87 mg±0.29 mg SD; calculated from a ubiquitous sample of minima workers taken from the 3 colonies for weighing, n = 60) was released in the foraging arena with free access to the nest. The mesh partition enabled medium workers to antennate the brood behind it, and minima workers to walk through and care for them. The side of the brood-containing nest chamber was alternated between assays. Familiarization time was 18 hours, after which the number of ants that aggregated in each chamber was counted, and 0.5 g of fungus, freshly collected from the same colony as the ants, was added in the foraging arena. The unfavorable low humidity (∼50%) in the open box prompted ants to relocate the fungus inside the more humid nest. An assay was finished when workers relocated all fungus from the foraging arena into the nest. Afterwards, the fungus in each nest chamber was weighed to the nearest 0.1 mg. A total of 12 replicates were performed. Due to a limited number of available laboratory colonies, the experimental series as well as the next series described below were performed with workers, brood and fungus from a single colony. It is therefore unclear if the outcome of this experiment can be considered as representative for the response of other colonies.

The second experimental series was aimed at evaluating whether the deposition of fungus at sites containing brood was actually a direct response to the brood presence. In the previous series fungus-carrying workers may have found the brood pile by following, for instance, pheromone markings left by ants as they aggregated in the brood containing chamber or by colony odors left on tunnel or chamber walls. By shortening the time span within which pheromones or colony odors could accumulate and offering brood at one spot in a relatively spacious chamber, fungus accumulation around this spot should be considered as a direct response to the brood presence. This may suggest that cues originating from the brood (i.e., pheromones, released CO_2_) could also modulate the response threshold to engage, for instance, in digging, which might be relevant for chamber emergence and for the digging experiments described below.

The experimental set-up offered a nest site consisting of a single, spacious chamber (a line drawn on the chamber floor virtually divided the chamber in two halves), and ants were allowed to familiarize with it for a shorter period (Experiment 4, Fig. 1d, right). A similar round plastic arena (diameter 15 cm, height 1 cm) was used as a nest site, which was only partly filled with plaster forming a straight wall. The two mesh enclosures were fastened at opposite ends into the plaster wall. Entering workers could easily move across the single chamber and reach the enclosures. The plaster was remoistened with 5 ml of demineralized water and 20 freshly collected pupae were placed in one of the enclosures. The side of the brood-containing mesh was alternated between assays. Then a foraging arena (an open plastic box, 19×19×9 cm) was connected to the nest site. At the beginning of each assay 50 medium and 10 minima workers were released in the foraging arena. After a 2 h familiarization period the number of workers present in each chamber ‘half’ was counted. Then 0.5 g fungus was placed into the foraging arena, but not in a single large piece as in the former series. It had been divided into 20 equally-sized, transportable pieces. The nest side to which the first 10 fungus pieces were relocated was noted and 13 replicates were performed.

### (c) Chamber excavation as a response to the presence of brood and fungus

In order to evaluate whether the presence of brood or fungus at a site leads to the excavation of a chamber around them, workers' digging activity was quantified in a binary-choice experiment offering two suitable digging sites (temperature ∼25°C, air humidity close to saturation). Two different experimental series were performed (Experiments 5 and 6, Fig. 1e), presenting either brood or brood plus fungus as stimuli.

#### Brood stimulus

In the first series (Experiment 5), the brood was offered as a stimulus at one of the nest excavation sites, because of the observed preferences for brood relocation (further details in the Results). The setup for the first series was as follows. For each assay two round nest sites (diameter 15 cm, height 1 cm) were filled with moist clay (water content 18%) and a single tunnel (4×1×0.5 cm) was cut out of the material in each (Fig. 1e). Twenty pupae were placed in a preformed tunnel of one of the digging sites (alternated between assays). The digging sites were connected with each other and the two-box setup described in Experiment 1. The use of two separate nest sites connected via tubing, instead of a Y-shaped tunnel cut out in a single nest site allowed excavation only to occur at the two small tunnels, and therefore enabled a clear quantification of the emerging structures. At the beginning of each assay a group of 100 workers was released in the foraging arena, and from there workers had access to the soil deposition site and both digging sites. After 24 hours, the amount of excavated clay in each nest site was quantified to the nearest 0.1 g and the excavated volume (cm^3^) calculated (1 cm^3^ = 1.8 g of clay). Fifteen replicates were performed, 5 replicates per colony.

#### Brood and fungus stimulus

In the second series (Experiment 6), we quantified the effect of a subsequent fungus deposition at the digging site on chamber emergence. The setup was identical to that used in the previous experimental series, with 20 pupae placed in one tunnel and a worker group of 100 ants released in the foraging arena. Then, 1 hour after workers started to dig and excavated clay pellets were deposited in the connecting tube, 0.5 g of freshly collected fungus was placed in the foraging arena. The low humidity there (∼50%) caused the ants to relocate the fungus into the more humid nest sites (values close to saturation, 99.9%). After 24 hours, fungus and excavated material at each digging site were quantified to the nearest 0.1 mg and 0.1 g respectively, and the excavated volume was calculated. A total of 15 replicates were performed, 5 per colony.

### (d) Shape of excavated structures

Even when comparable amounts of soil are excavated from digging sites, the shape of the resulting structure might vary from an intricate tunnel system to a more chamber-like, round cavity. To obtain a measure of the circularity of the excavated structures, i.e. of their cavity-like shape, their form factor was calculated. The form factor is the ratio of the area of an object to the area of a circle with the same perimeter as the object [48], as follows:
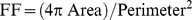



The form factor varies from 0 to 1; the higher the value, the more circular the structure. To determine the area and the perimeter of the excavated structure, a plaster cast of the excavation was made of both digging sites at the end of each assay (for both experimental series), which were digitized with a scanner. Then, area and perimeter were measured using the software ImageJ (version 1.44p, National Institutes of Health, USA) and the form factor was calculated. Because of the offered preformed nest tunnel and entrance hole, the starting form factor of each excavated structure was not 0, but 0.28, the baseline from which the shape of the excavated structures could develop.

## Results

### (a) Determination of relocation preference for brood or fungus

**Figure 2:**
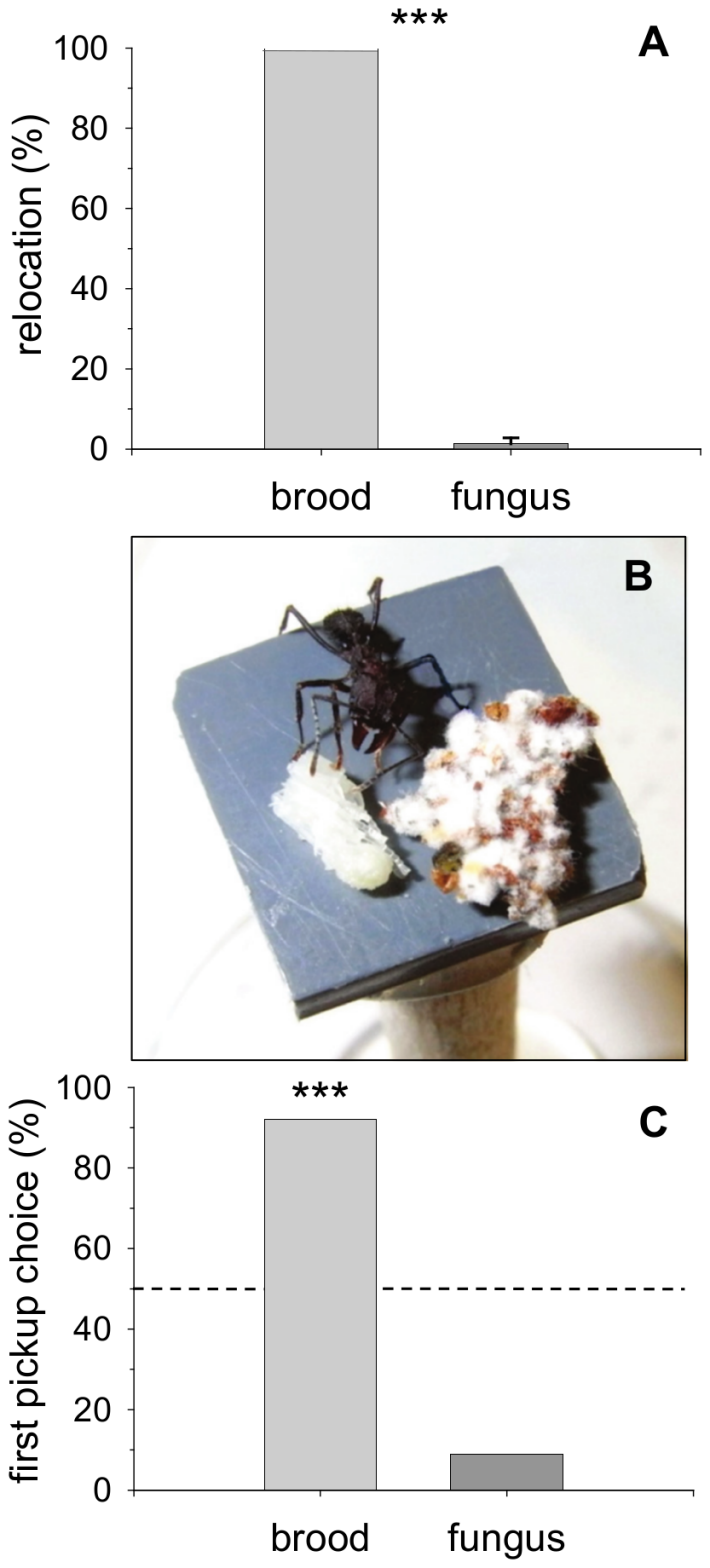
Brood and fungus relocation experiments. (a) Experiment 1: Relocation of items from cold stress (10°C) by a worker group after 24 h, presented as percentage of the total offered amount (20 pupae and 0.5 g fungus) (median ± 25–75% percentiles), ***p<0.001 (Analysis of colony effects: Kruskal-Wallis-Test; Brood relocation; H = 0.0; p = 1; n = 5; Fungus relocation; H = 0.66; p = 0.72; n = 5; n.s.; no colony effects found). (b) Experiment 2: Relocation of items from desiccation by single workers: an *A. lundi* worker encounters a pupa and a piece of fungus on an experimental platform (1.5×1.5 cm). (c) Score of first item relocated, expressed as percentage of total observations (n = 101), ***p<0.001 (Analysis of colony effects: Fisher's Exact test for 3×2 contingency tables; p = 0.24; no colony effects found).

No relocation of brood or fungus was observed before the cooling of the nest (Experiment 1). After 24 hours, the proportion of relocated brood was significantly higher than that of relocated fungus (Fig. 2a; Wilcoxon matched pair test; T = 0.00; Z = 3.41; p<0.001; n = 15). In each of the 15 assays, ants relocated all live pupae (100%) into nest site 2, but only a median of 1.36% (25–75% percentiles = 0–2.78%) of the original 0.5 g fungus mass (0.0068 g; 25–75% = 0–0.0139 g). In 4 of the 15 assays no fungus at all was relocated. Single ants also showed a preference for brood relocation when a pupa and a piece of fungus were offered side by side (Fig. 2b, Experiment 2). In 92 (91.1%) of 101 observations, the first item picked up was brood (binomial test; p<0.001; Fig. 2c). The mass of a brood item (7.2±0.18 mg, mean ±SE), and that of a fungus piece (13.9±0.33 mg, mean ±SE) equals a burden ( = (ant mass+load mass/ant mass); load size expressed in relative terms) of ∼2–3.5. Leaf-cutting ants can carry a burden of up to 7.5 when they forage leaf-fragments [49], indicating that both items were easily transportable in our experiment. Upon discovering both items on the platform, ants were observed to antennate them with slightly opened mandibles and protruded labium, and then to pick up one item and to carry it into the nest. The time lapse between the first and second item being picked up was measured in 60 of the performed 101 observations (fungus as the second item picked up: n = 53; brood as the second item picked up: n = 7). Fungus was picked up 47.7 s (±29.3 SE) after the brood item, and brood was picked up 92.2 s (±20.2 SE) after the fungus. Never was the second item not picked up by another worker, indicating that both brood and fungus pieces were healthy and undamaged.

### (b) Brood as a cue for fungus relocation

**Figure 3:**
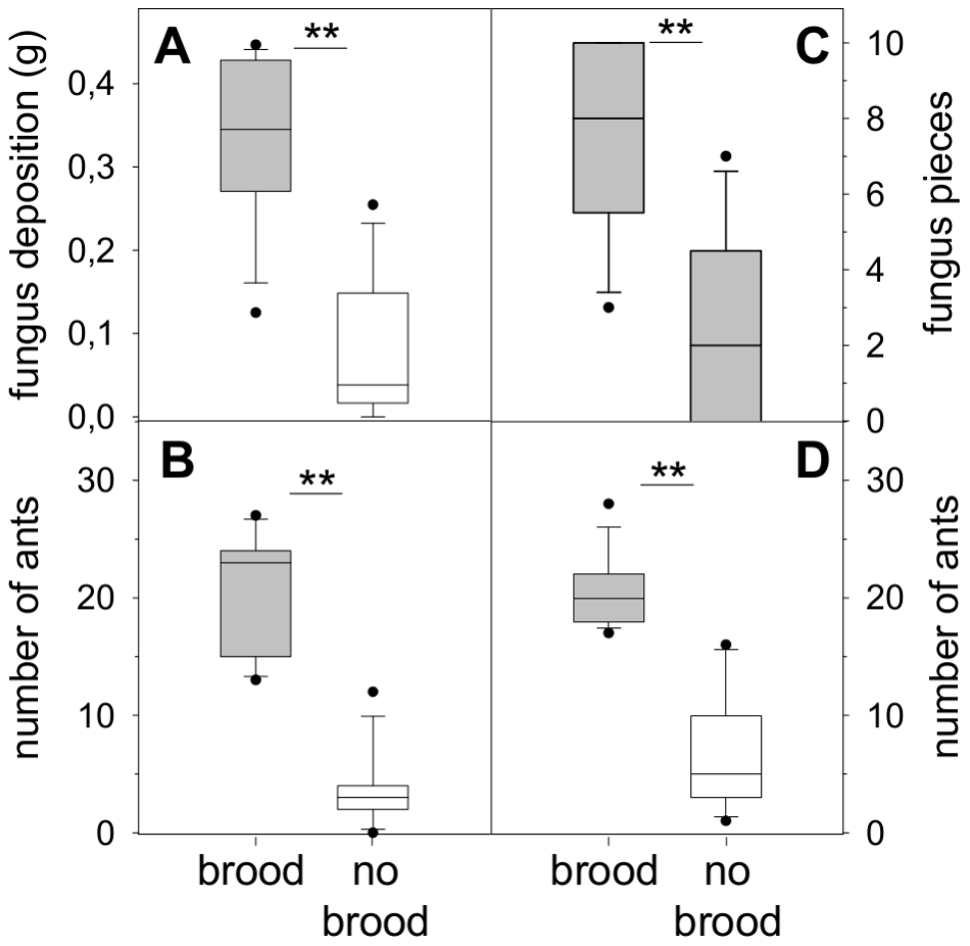
Fungus relocation experiment in non-digging setup. (a) Experiment 3: Fungus deposition in chambers, only one containing brood (n = 12). (b) Number of *A. lundi* workers in chambers with and without brood (n = 12). (c) Experiment 4: Fungus deposition at brood and empty side (n = 13). (d) Number of workers at brood and empty side (n = 13). Boxplots: median ± 25–75% percentiles, min max values and outliers, **p<0.01.

When workers could choose to relocate fungus into an empty nest chamber or one with brood, the majority of the relocated fungus was deposited in the chamber containing the brood (Experiment 3; Fig. 3a; Wilcoxon matched pair test; T = 3.0; Z = 2.824; p<0.01; n = 12). The median fungus deposit in the brood chamber was 0.345 g (25–75% = 0.276–0.428 g) and 0.038 g (25–75% = 0.017–0.129 g) in the empty chamber. Most of the pieces were placed side by side on the chamber floor, and rapidly filled the available space in the brood chamber. It is therefore likely that further fungus relocation was not possible because of lack of space, and it was shifted to the alternative, brood-less chamber. Time differences of occurrence of the first fungus deposit in the chambers seem to support this view (in the brood chamber; median = 7.5 min; 25–75% = 5.5–11 min; in the empty chamber; median = 17 min; 25–75% = 13–27.5 min; Wilcoxon matched pair test; T = 5.000; Z = 2.667; p<0.01; n = 12). In addition, in 10 of the 12 assays the very first piece of fungus relocated inside was deposited in the brood chamber. A difference in the magnitude of worker aggregation in the two chambers was also observed. Significantly more ants were located in the brood chamber before the fungus was placed in the foraging arena and relocation took place (Fig. 3b; workers in brood chamber; median = 23; 25–75% = 16–24; workers in empty chamber; median = 3; 25–75% = 2–4; Wilcoxon matched pair test; T = 0; Z = 3.06; p<0.01; n = 12).

Even when a nest site with one big chamber instead of two small separate ones was offered (Experiment 4; Fig. 3c), and the familiarization period was shortened from 18 to 2 h, significantly more fungus pieces were deposited on the brood side of the chamber (Wilcoxon matched pair test; T = 5.5; Z = 2.63; p<0.01; n = 13). Of the 130 deposited pieces (first 10 of each assay, n = 13), 97 were deposited on the brood, 33 on the empty side of the chamber. There also was a significant skew in ant aggregation in favor of the brood side. A median of 20 ants (25–75% = 18–21) were present on the brood side while a median of 5 ants (25–75% = 3–8) were present on the empty side (Fig. 3d; Wilcoxon matched pair test; T = 0; Z = 3.18; p<0.01; n = 13).

### (c) Chamber excavation as a response to the presence of brood and fungus

**Figure 4:**
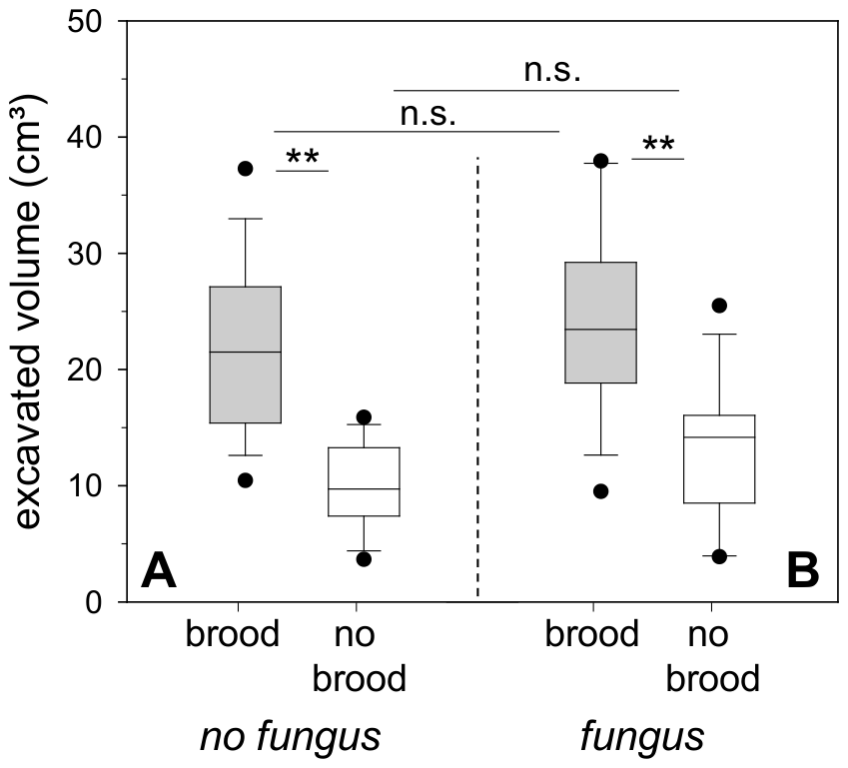
Digging activity (excavated volume) at nest sites with and without brood after 24 h (median ± 25–75% percentiles, min max values and outliers, n = 15). **p<0.01; n.s. = not significant, p>0.05 (a) Experiment 5: Digging activity with brood only (Analysis of colony effects: ANOVA; brood; F = 1.91; p = 0.19; n = 5; no brood; F = 0.25; p = 0.79; n = 5; n.s.; no colony effects found) (b) Experiment 6: Digging activity with brood and fungus (Analysis of colony effects: ANOVA; brood and fungus; F = 0.64; p = 0.54; n = 5; fungus; F = 0.22; p = 0.8; n = 5; n.s.; no colony effects found).

When given the possibility to either dig at a nest site with brood or at an empty one with no fungus present, ants excavated at both sites, but more material was removed from the nest site with brood (Experiment 5; Fig. 4a; Wilcoxon matched pair test; T = 3.0; Z = 3.24; p<0.01; n = 15). The median excavated volume at the brood nest site was 21.5 cm^3^ (25–75% = 15.39–27.11 cm^3^; min-max = 10.44–37.28 cm^3^) and at the site without brood 9.72 cm^3^ (25–75% = 7.39–13.28 cm^3^; min-max = 3.67–15.89 cm^3^). Workers were observed to continue with excavation even when the experiment was stopped after 24 hours, although the space created around the brood looked more than sufficient to house both workers and brood. In fact, the brood pile took up very little space, and 10 piled pupae, as an example, occupy a mean area of 0.95 cm^2^ (±0.25 SD) on the floor and a volume of less than 1 cm^3^.

**Figure 5:**
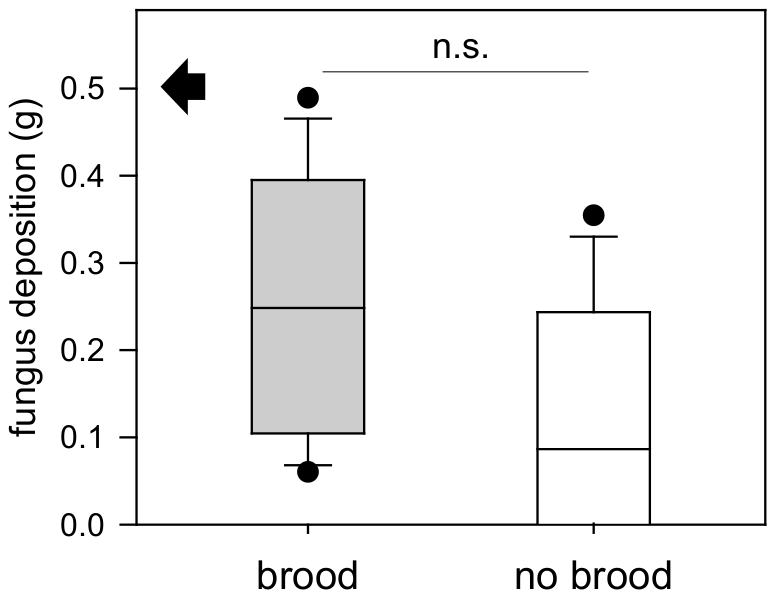
Experiment 6: Fungus deposition at nest sites (median ± 25–75% percentiles, min max values and outliers, n = 15); black arrow indicates maximum possible fungus deposit; n.s. = not significant, p>0.05 (Analysis of colony effects: Kruskal-Wallis test; brood site; H = 1.94; p = 0.38; n = 5; non-brood site; H = 2.2; p = 0.33; n = 5; n.s.; no colony effects found).

Regarding the additional relocation of fungus inside excavated nest sites with and without brood, the amount of relocated fungus did not differ significantly between the sites (Experiment 6; Fig. 5; brood side: median = 0.248 g; 25–75% = 0.105–0.395 g; min-max = 0.060–0.489 g; non-brood side: median = 0.087 g; 25–75% = 0.001–0.244 g; min-max = 0–0.355 g; Wilcoxon matched pair test; T = 29; Z = 1.76; p>0.05; n = 15), although in 3 of the 15 performed assays no fungus at all was deposited at the non-brood nest site. As in the previous series, digging activity concentrated at the site containing brood and relocated fungus, and a significantly higher volume was excavated there (Experiment 6; Fig. 4b; brood side; median = 23.44 cm^3^; 25–75% = 18.83–29.22 cm^3^; min-max = 9.5–37.94 cm^3^; non-brood side; median = 14.17 cm^3^; 25–75% = 8.5–16.06 cm^3^; min-max = 3.89–25.5 cm^3^; Wilcoxon matched pair test; T = 10.0; Z = 2.84; p<0.01; n = 15), despite the equal fungus deposit in both. Brood and fungus pieces were placed together; sometimes pupae were placed on top of the fungus.

**Figure 6:**
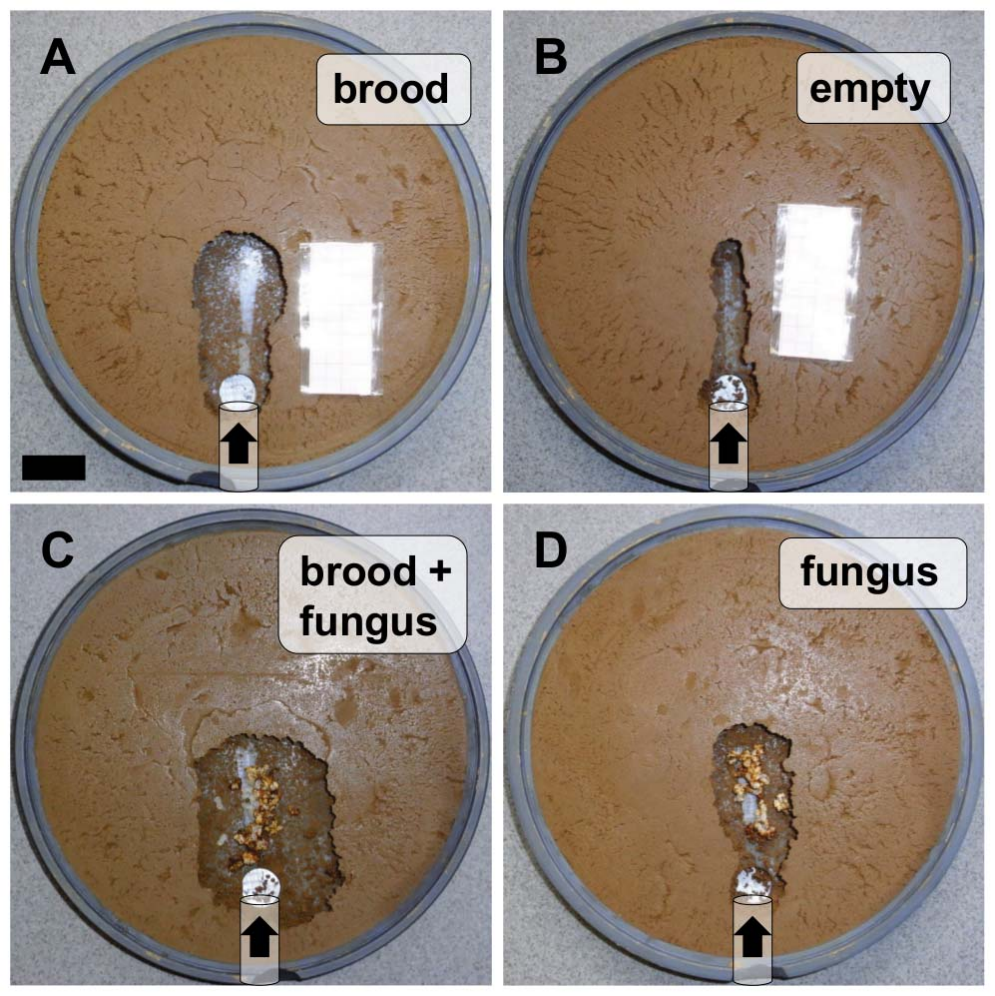
Pictures of digging sites at the end of the experiments. Experiment 5: (a) Brood. (b) Empty. Experiment 6: (c) Brood and fungus. (d) Fungus. Black bar = 2 cm; black arrows indicate the direction of entering ants.

Although the more voluminous fungus pieces are expected to take up more space than pupae, the volume excavated when fungus was present was not higher, but similar to that from the series with only brood present (Fig. 4; brood vs. brood and fungus: Mann-Whitney U Test; U = 97.0; p>0.05; n = 15; empty vs fungus: Mann-Whitney U Test; U = 76.0; p>0.05; n = 15). Examples of the excavated nest sites of all 4 different types (i.e., brood, empty, brood+fungus and fungus) are presented in Fig. 6.

### (d) Shape of excavated structures

**Figure 7:**
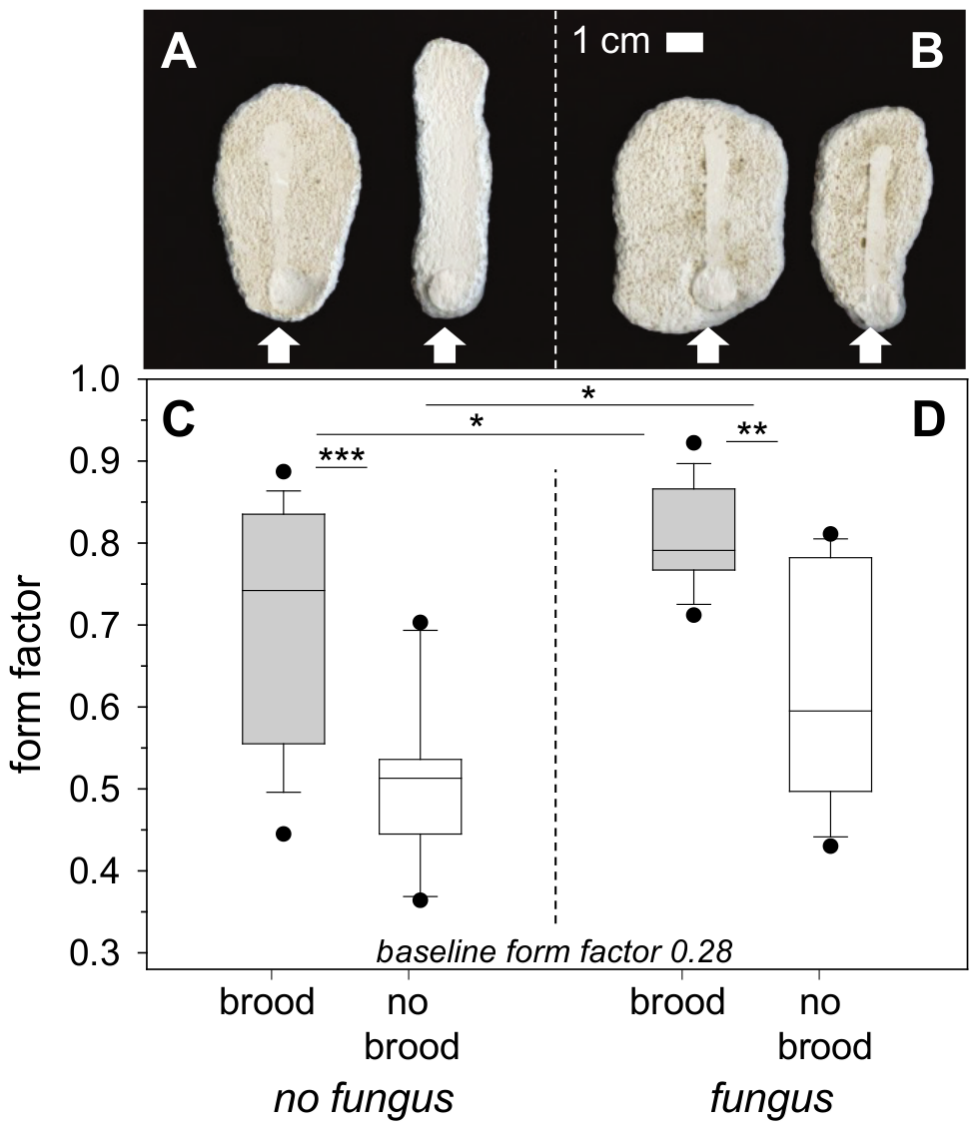
Evaluation of excavated shapes. (a) Plaster molds of excavation, brood only series (Experiment 5), Arrows indicate the direction of entering ants; view from below. (b) Plaster molds of excavation, brood and fungus series (Experiment 6). (c) Calculated form factor of brood only series (Analysis of colony effects: ANOVA; brood site; F = 0.71; p = 0.5; n = 5; non-brood site; F = 0.14; p = 0.87; n = 5; n.s.; no colony effects found). (d) Calculated form factor of brood and fungus series (Analysis of colony effects: ANOVA; brood and fungus site; F = 1.24; p = 0.32; n = 5; fungus site; F = 1.67; p = 0.23; n = 5; n.s.; no colony effects found). The y-axis starts from a baseline form factor of 0.28 (preformed structure: entrance hole and tunnel) (median ± 25–75% percentiles, min max values and outliers, n = 15), *p≤0.05; **p<0.01; ***p<0.001.

In the presence of brood, the shape of the excavated structure was more circular and therefore more chamber-like than without brood (Experiment 5; Figs. 7a and 7c; Form Factor (FF) brood site; median = 0.74; 25–75% = 0.56–0.84; FF empty side; median = 0.51; 25–75% = 0.45–0.54; Wilcoxon matched pair test; T = 0.00; Z = 3.41; p<0.001; n = 15). The lower form factor for the non-brood site indicates a very high ratio of perimeter to area of the structure, i.e., a more tunnel-like shape.

In the series with additional fungus relocation, the excavated shapes at the nest sites containing brood were again significantly more circular and chamber-like than at the non-brood site (Experiment 6; Figs. 7b and 7d; FF brood site (fungus present); median = 0.79: 25–75% = 0.77–0.87; FF non brood site (fungus present); median = 0.60; 25–75% = 0.50–0.78; Wilcoxon matched pair test; T = 9.0; Z = 2.90; p<0.01; n = 15), even though a similar amount of fungus, which is known to influence the shape of a chamber in accordance to its volume [45], was relocated to both sites. When comparing the shapes excavated at the brood and the non-brood site between the two different series, i.e., with or without additional fungus relocation, it was evident that the presence of fungus had a positive effect on the roundness of the excavated shape. The excavated structures at nest sites with both brood and fungus were rounder than at those with only brood (Figs. 7c and 7d; Man-Whitney U Test; U = 62.0; p<0.05; n = 15), and the excavated structures at sites with only fungus were rounder than at those with neither brood nor fungus (Man-Whitney U Test; U = 61.50; p<0.05; n = 15), with the least circular shapes being excavated at the latter, empty nest site.

**Figure 8:**
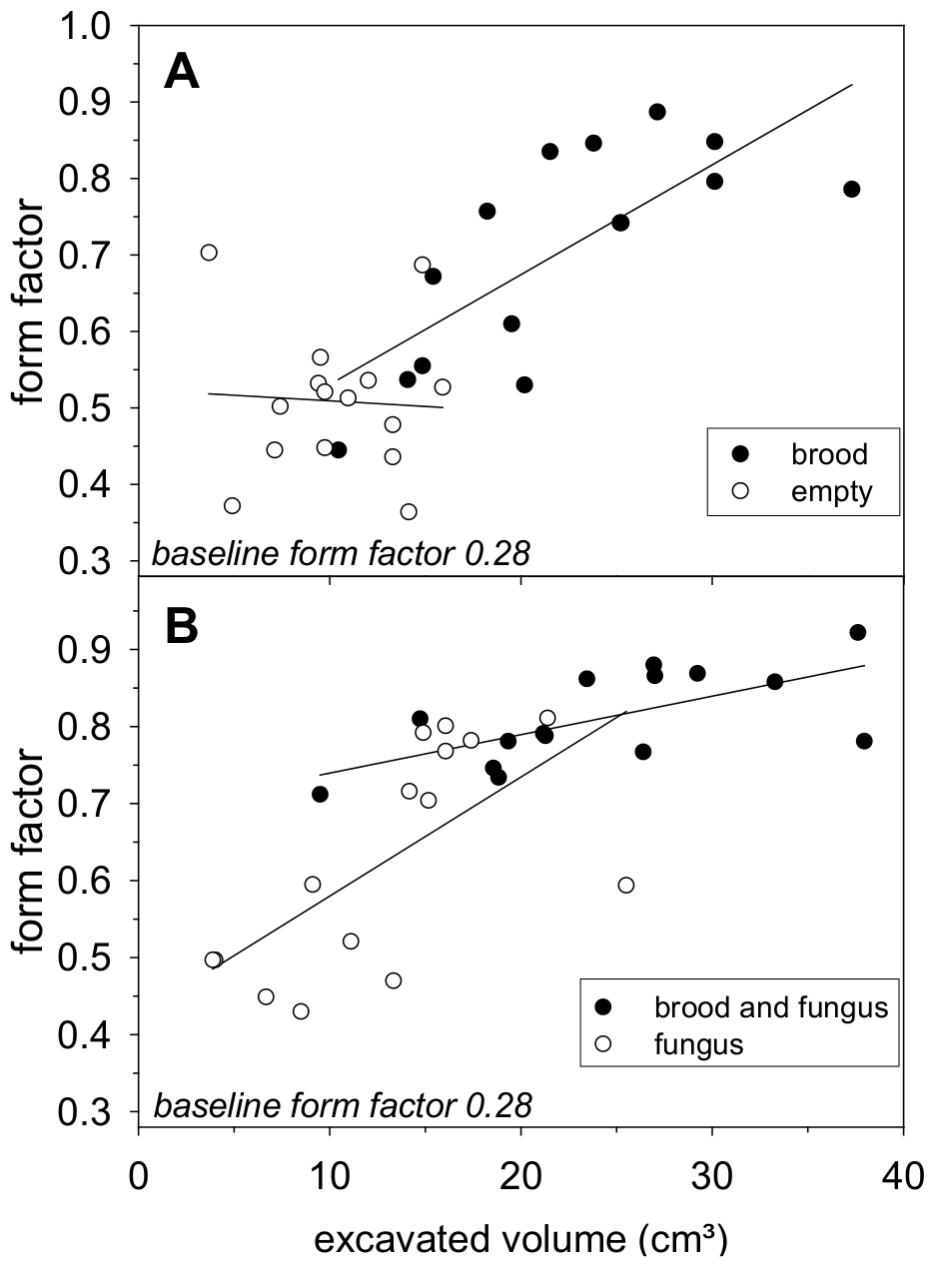
Relationship between the digging activity, measured as the excavated volume (x-axis), and the excavated shape, expressed as the form factor (y-axis). (a) Experiment 5: brood only series. Closed circles: brood; y = 1E-6x+0.387; open circles: no brood (empty tunnel); y = 1E-07x+0.523. (b) Experiment 6: brood and fungus series. Closed circles: brood and fungus; y = 5E-07x+0.689; open circles: fungus; y = 2E-06x+0.425; y-axis starts from a baseline form factor of 0.28.

A regression analysis was performed to evaluate whether the circularity of the excavated shapes depended on the excavated volume, i.e., the more material excavated, the rounder the resulting structures (Figs. 8a and 8b). Only at the nest sites with items (brood, fungus, or brood and fungus) was there a positive correlation between excavated volume and circularity (brood; r^2^ = 0.56; p<0.01; fungus; r^2^ = 0.42; p<0.01; brood and fungus; r^2^ = 0.42; p<0.01). When the nest site was empty, the excavated shapes did not increase in circularity (Fig. 8a; empty; r^2^ = 0.04; p>0.05), although excavation ranged from 4–15 cm^3^. At the sites with items, however, an increase of 11 cm^3^ of excavated space led to a clear increase in circularity.

## Discussion

Traditionally, abiotic factors such as humidity and temperature gradients have been described as the local stimuli workers use to choose a place for rearing their brood and fungus [37], [38]. The excavation of new fungus chambers at locations with suitable environmental conditions would therefore ensure proper development of the fungus and brood. However, the questions arises whether such suitable conditions suffice to trigger the excavation of a new chamber in advance, without the presence of fungus and brood at the spot. It is known that the symbiotic fungus, when relocated into a preformed, round chamber providing insufficient space, triggers the enlargement of the chamber in laboratory colonies of *Acromyrmex lundi* [44], [45]. Workers excavated around the relocated fungus, thus extending the size of the initial chamber to accommodate all fungus. Without the presence of fungus, workers only excavated tunnels [44], [45]. While chambers can be enlarged when its content outgrows the offered space, probably by using the fungus as a template, there appears to be a maximal chamber size, so that at a given time new chambers need to be excavated. The abandoning of chambers because of unsuitable climatic conditions or the presence of contaminants should also go along with the excavation of new chambers.

Our results extend the knowledge about the emergence of nest structures by showing that the emergence of a new chamber can be triggered by the presence of brood at a site, with only tunnels being excavated at an alternative location without brood. These results are consistent with the hypothesis that nest chambers are not excavated in advance. Suitable microclimatic conditions alone do not appear to be sufficient to trigger chamber excavation. Based on the present results we propose a density-triggered mechanism of cavity excavation by which chambers emerge as functional structures when brood and fungus, i.e., the items that are expected to be stored in these cavities, are relocated and present at a given spot. Brood and fungus appear to serve as cues that attract workers and draw them away from other, environmentally suitable digging sites, thus leading to a high worker density around these items. Even though *A. lundi* inhabits nests with only a few chambers [11], usually two to three, and sometimes only one, their shape is similar to that of other leaf-cutting ant species [6]. We suggest that the proposed mechanism of chamber emergence and nest enlargement via relocated items is likely to be a common local mechanism that could also underlie the growth of the multi-chambered nests of *Atta* leaf-cutting ants, irrespective of their total number of chambers.

### (a) Determination of relocation preference for brood or fungus

*A. lundi* workers showed a significant preference to relocate brood before relocating fungus, when exposed to a low temperature (Experiment 1) known to impair brood and fungal development [36]. This tendency to remove more brood than fungus was also observed in *Acromyrmex heyeri* workers [37], which were in the process of carrying brood or fungus and exposed to a temperature of 10°C. As a result they relocated more brood (40%) than fungus pieces (20%). In our experiments, it was surprising that so little fungus (in some assays none at all) was relocated to temperatures above 20°C. There was no apparent indication that during the experiment the fungus was damaged or died, because it would have been removed to the foraging area (foraging arena and soil deposit site), as observed in other experiments. While it could be argued that some of the fungus was infected with pathogens and therefore was not relocated, the uniformly low fungus relocation would then imply that all the fungus collected from three different colonies and different gardens had been infected. It seems unlikely that fungus mortality or a pathogen infection was the reason for the reduced rate of fungus relocation in our assays. It is possible that the higher relocation rate of fungus in *A. heyeri* [37] was due to workers already carrying the fungal pieces when exposed to the low temperatures, while in our experiments ants needed to cut free a piece of fungus first. The low temperature itself did not negatively influence the activity of the ectothermic ants, because the process of brood relocation was not affected and occurred completely.

There are other possible reasons why brood were relocated before the fungus. From an energetic perspective brood might be more costly to produce than fungus. In the laboratory we could observe that development from eggs to pupae took several weeks while the ants managed to create a new fungus garden (∼1.3 l) in a week with ad libitum feeding. The second reason could be that there were microscopic traces of fungus left on the pupae we used in the experiments, so that fungus would be indirectly relocated with the brood. It is known that *Acromyrmex* pupae usually have a mycelial cover, not only from being embedded in the fungus garden, but also because workers actively plant these covers on the brood [47]. We removed any visible traces of fungus mycel from the pupae before the experiments, but there might have been microscopic traces left. It is unknown whether these traces would have been enough for the ants to start a new fungus garden. However, all fungus was still relocated into the nest site in Experiment 2, if only after the brood had been removed. If a mycelial cover influences the ants not to relocate fungus, they also should not have done so in Experiment 2. Third, brood might be easier to handle than fungus. Cutting out a piece of fungus takes time, which may lead ants to transport first the easily transportable items in a situation of rapidly changing environmental conditions. It has been reported that in a partly flooded field nest of *Atta sexdens*, part of the colony brood had been deposited in an upper, safe nest chamber, but none of the fungus gardens were relocated there [3]. That the time-consuming fungus removal was not the reason for the workers preference for brood relocation could be demonstrated when fungus was offered in small, easily transportable pieces (Experiment 2). Yet in over 90% of the pickup decisions, workers favored brood to be relocated first from desiccation. On the other hand this could indicate that brood is more prone to desiccation than fungus. However, the air humidity inside the clay nest sites in Experiment 1 was close to saturation, yet brood relocation was also preferred. Offered pupae and fungus pieces also differed in mass, a piece of fungus was twice as heavy as a pupa. Although the masses are in the range of naturally foraged loads [49] it could still be energetically more advantageous to carry a pupa. When ants picked up an item on top of the platform they were never observed to lift one item, put it down again and picking up the other item, as if the chosen item was too heavy to transport, a mechanism that is thought to lead to size-matching between carriers and their leaf fragments during foraging [50]. Because ants always chose only one item and relocated it, we rule out differences in mass as a reason for the preferred brood relocation.

The motivation of ants to relocate exposed items from the foraging arena into the nest likely differs from that to relocate them within the nest. A stronger motivation to protect the items against exposition to the unsuitable outside environment may have been the reason for the complete relocation of fungus in the second series, yet brood were removed before the fungus. This seems to indicate that early brood removal in Experiment 1 was not just due to the physical restraints of the interconnected fungal mass on transportation. We therefore argue that *A. lundi* leaf-cutting ants seem to have a preference for brood relocation from sites having unsuitable conditions, and suggest that the observed pattern of relocation from the foraging arena (Experiment 2) reflects a transportation pattern that is also expected to occur within the nest. This was the reason why brood was tested as a possible trigger for chamber emergence in the digging experiments performed later.

### (b) Brood as a cue for fungus relocation

Because of the association of brood and fungus in nest chambers [46], [47], it was hypothesized that when brood is removed to other places in the nest, fungus pieces should be also relocated to these spots. The results of the fungus relocation experiments (Experiments 3 and 4) are in accordance with this hypothesis. Workers deposited more fungus at a location containing brood, irrespective whether the brood occurred in a separate chamber or at one side of a single chamber, suggesting that workers directly responded to the presence of brood. It remains an open question whether brood also directly influences the intensity of digging activity of those workers, which would later engage in the excavation of a chamber around it. Fungus would benefit from being relocated to a brood side because they have the same microclimatic demands on temperature and humidity. When workers of *A. heyeri* relocating brood and fungus could chose a deposition site along a temperature gradient, they selected temperatures from 21 to 25°C for both [37], values that are known to ensure optimal fungal growth [36]. As a consequence, the presence of brood as a spatial cue for fungus deposit at such a site should also benefit fungal development.

Brood likewise might benefit from being surrounded by fungus. There could be several reasons: reduction of water loss through its not yet hardened cuticle, better insulation against temperature fluctuations, or reduction of risks of pathogen transmission by the barrier created by the fungus between brood and chamber floor. The experiments also highlighted how attractive brood was to workers, which will be of importance for our proposed mechanism of chamber excavation explained below. On average, 5–6 times more ants aggregated at the nest site that contained brood, so ant density clearly increased at this spot. Ant density likely plays an important role in nest excavation because high density is thought to stimulate workers to dig [25], [51]. Such a density-triggered excavation behavior would result in the enlargement of a nest whenever the population increases, thus leading to a temporary increase in ant density, which would decline, once more space has been excavated. The fact that the size of many ant nests correlates with the number of ants inhabiting it, and larger colonies inhabit larger nests [25], [52], support this hypothesis. Increased ant aggregation at brood deposition sites could result in a higher number of ants excavating at such a site, or an increased per capita excavation activity of workers, variables that were not quantified in our study.

### (c) Chamber excavation as a response to the presence of brood and fungus

The results of the digging experiments indicate that brood presence leads to a spatial shift of digging activity towards a nest site containing brood, thus resulting in the excavation of rounder, more chamber-like shapes at this site (Experiment 5). It is likely that the presence of brood at the digging site caused a higher aggregation of workers at the site, as demonstrated in the fungus-relocation experiments mentioned in the previous section (Experiments 3 and 4), and that more workers engaged in digging there. Due to the opacity of the digging material, the number of ants engaged in digging could not be directly counted because parts of the excavation occurred under a layer of clay. Since significantly more ants aggregated at the brood site than at the site without brood in the fungus-relocation experiments (Experiments 3 and 4), it seems reasonable to infer that the effect of brood on worker aggregation should have been similar in the digging experiments. As a consequence, more workers present at a nest site with brood would lead to a higher excavated volume and a rounder cavity. In addition, brood could directly influence the intensity of digging in individual workers. While there are no comparative measurements of chamber shapes in field nests of leaf-cutting ants, nest tunnels are by definition long and narrow, meaning they have a horizontal cross-section with higher perimeter-to-area ratio than chambers, which are spherical and not lobed, in accordance with our results.

Regarding the hypothetical excavation of new nest chambers in advance, triggered solely by a suitable microclimate, it is important to note that the environmental conditions offered at both nest excavation sites during our experiments were identical, and suitable for brood and fungus development. If environmental factors were the only cues ants use to decide where to initiate the excavation of a chamber, the shapes of the excavated structures should have been similar to one another. However, at the site with no brood or fungus present, ants excavated structures with shapes that resembled the preformed offered tunnel, and typically concentrated their digging at the tunnel tip. This was likely due to a disparity of worker number in favor of the site containing brood.

As previously indicated, it is known that the presence of fungus leads to chamber excavation [44], [45]. Workers excavated a cavity around the relocated fungus that was slightly bigger than the actual fungal structure and also shaped according to its proportions. When the fungus grew, so did the size of the chamber. Fungus in this regard was used as a dynamic template for the size and shape of the nest chamber. The use of brood as a template to shape the nest is known in the ant *Leptothorax tuberointerruptus* that inhabits very simple nest cavities. The amount of brood deposited in the middle of a worker cluster acts as a template for the erection of a surrounding wall that embodies all colony members [24]. In our experiments however, considering the large amount of space being excavated around brood, and the brood pile often being located not centrally in the excavated structure but to the side, it seems unlikely that the presence of brood alone is used as a template for chamber excavation.

Using fungus as a template is likely not the only variable involved in the determination of chamber size in leaf-cutting ant nests. The cavities excavated in our experiments with both brood and fungus (Experiment 6) were not of equal size, although an equal amount of fungus, i.e., a template of comparable size, had been deposited at both nest sites. Excavation at the brood (and fungus) site was higher than at the site with only fungus, probably because a higher number of workers were already present at the site with brood. Fungus relocation in this digging experiment did not follow the brood deposition, contrary to the expectations based on the relocation experiments with plaster nests (Experiments 3 and 4). This was probably due to the lack of space in the digging experiments, in which only small tunnels were offered. These results indicate that leaf-cutting ants, instead of letting the fungus die, relocate it to other suitable sites that they otherwise might not have chosen. The observed excavation of rounder shapes at the nest site containing only fungus emphasizes that fungus deposition triggers chamber emergence by influencing workers' digging activity in a way that rounder, more chamber like structures are excavated, even without brood.

The presence of brood and fungus concentrates excavating workers at the spot, leading to an evenly spread digging activity around it. This is highlighted by the existing correlation of excavated volume with the circularity of the excavated shapes only when at least either brood or fungus was present. A nest excavation site without relocated items might increase in excavated volume, but, likely because the ant workforce is not concentrated at a particular spot while digging, a less round and more tunnel-like structure is expected to emerge. Two mechanisms influencing ant aggregation, and therefore local ant density during nest digging, were recently described in leaf-cutting ants: a short-range vibrational signal, and even the presence of excavated soil pellets. Leaf-cutting ants stridulate while excavating, which attracts nearby workers to the digging site [53]. This effect could have led to an amplification of ant aggregation (and excavation) at the nest site where brood had been placed and workers had already started excavating and stridulating. The concentration of digging activity at a particular spot would be further guided by the presence of freshly-excavated pellets, deposited close to the excavation site, because they significantly influence the workers' decision where to start digging [54]. While each mechanism could work on its own to lead to ant aggregation influencing digging activity, they may also have had additive effects, as follows. Workers may have initially been attracted to one nest site because of brood, and started to dig because of the increased ant density there. The resulting presence of stridulating workers may have attracted more ants to the site, which led to further excavation there and the accumulation of soil pellets. This prompted even more workers that were present to engage in digging, leading to the significant difference in the volume and roundness of the excavated structures we observed.

### (d) Mechanism of chamber emergence

We suggest the following mechanism underlying the emergence of a new chamber in a leaf-cutting ant nest. Because of space requirements or unsuitable conditions, brood and/or fungus are expected to be relocated from an existing nest chamber to a more suitable location in a nest tunnel. Due to the attractiveness of both brood and fungus, workers aggregate at the site, thus leading to a local increase of ant density. Workers would then excavate in a density-dependent manner until sufficient space is generated, thus leading to the emergence of a new nest chamber. Digging activity is thought to positively depend on ant density [25], [55], [56]. Crowding may lower the behavioral threshold triggering digging, and lead to a higher number of digging workers at the site, with a larger space being excavated there. The results of the fungus relocation experiments (Experiments 3 and 4) suggest a direct response to the presence of brood, i.e., cues originating from the brood may influence other behavioral responses. Whether brood also has, for example, a stimulating effect on the per-capita excavation rate of workers remains to be investigated.

This postulated mechanism for chamber emergence could be solely based on the effect of worker density on digging activity, or also on an additional direct stimulating effect of brood on digging responses. In both scenarios, brood and fungus appear to act as ‘ant aggregators’ that concentrate the workforce at a suitable place in the nest, leading to the excavation of chambers with circular shapes. Therefore, not the mere presence of brood but the resulting increase in ant density would be the determining factor that leads to the excavation of more chamber-like shapes.

When ant workers are spread out across a large nest area with a wide digging face, only scattered digging sites are occupied and less circular shapes emerge. This effect was observed in a digging experiment with workers of the ant *Lasius niger*. The ants had access to a digging arena through a hole in the arena lid, without preformed space inside [55], [56]. They first excavated in a centrifugal way, creating a circular cavity that later became ramified as tunnels started to develop from the cavity wall. These results seem to indicate that chambers can emerge without the presence of any chamber items, contrary to the findings of our study and the arguments advanced above. It is important to indicate that *L. niger* workers had access to only one possible digging site inside the arena, so that all workforce was initially concentrated there, with the ants likely aggregating first at the entrance hole. The increased ant density at this spot, even without the presence of brood, likely stimulated more ants to engage in digging [51], so that a round structure was excavated. When the cavity grew, a decrease in ant density occurred, probably leading to a ‘competition’ of alternative, spatially-separated digging sites that attracted workers [57] and resulted in ramification of the excavated cavity and tunneling. As a consequence, it is likely that the initial cavity excavated by *L. niger* workers [55], [56] is not a functional structure aimed at generating nest space to house workers or brood, but resulted from the initial crowding effects and further dynamics of digging.

The importance of worker aggregation and the concomitant increase in ant density for the excavation of nest chambers, irrespective of the presence of brood, needs to be evaluated in further studies using for example single digging arenas in which available space and worker numbers, with and without brood items, should be manipulated. In a natural nest, ants should spread out across their nest space, as long as they do not encounter any stimuli triggering aggregation. A lower ant density would induce fewer ants to start excavating, with no concentration of digging activity at a particular spot. Therefore, chamber-like cavities should not be excavated there. We propose a distinction between calling an excavated space a chamber or a cavity, with the former term being used only when actual items usually housed in a chamber, i.e., brood, fungus or food, are present when the structure is excavated.

We suggest that the empty chambers that make up part of leaf-cutting ant nests [1], [2], [4], [5], [7], [20], [33], [34] were not excavated in advance, but rather initially excavated around relocated items, brood and fungus. They were found empty likely because of fungus decay, pathogen threat, or relocation of their contents to more suitable nest locations. Likely, it is energetically disadvantageous to engage in costly digging [58] in advance, before the actual need for chamber space arises, i.e., to excavate cavities that may not necessarily be used. Rather, we propose that chamber excavation is a self-organized process triggered by the aggregation of workers, i.e., by the increased ant density around relocated brood and fungus, which leads to a concentrated excavation at the deposition site and to the emergence of a chamber as a functional structure. Such a mechanism could hypothetically underlie the emergence of chambers in nests of other leaf-cutting ant species, and also in nests of non-fungus-growing ants that store brood or food, although the behavioral rules that lead to their species-specific architecture remain to be investigated.

## References

[1] Jonkman JCM (1980) The external and internal structure and growth of nests of the leaf-cutting ant *Atta vollenweideri* Forel, 1983 (Hym.: Formicidae) Part II. Zeitschrift für angewandte Entomologie 89: 217–246.

[2] Stahel G, Geijskes DC (1939) Über den Bau der Nester von *Atta cephalotes* L. und *Atta sexdens* L. (Hym. Formicidae). Revista de Entomologia 10: 27–78.

[3] Stahel G, Geijskes DC (1941) Weitere Untersuchungen über Nestbau und Gartenpilz von *Atta cephalotes* L. und *Atta sexdens* L. (Hym. Formicidae). Revista de Entomologia 12: 243–268.

[4] Moreira AA, Forti LC, Andrade APP, Boaretto MAC, Lopes JFS (2004a) Nest architecture of *Atta laevigata* (F. Smith, 1958) (Hymenoptera: Formicidae). Studies on Neotropical Fauna and Environment 39: 109–116 10.1080/01650520412331333756)

[5] Moreira AA, Forti LC, Boaretto MAC, Andrade APP, Lopes JFS, et al (2004b) External and internal structure of *Atta bisphaerica* Forel (Hymenoptera: Formicidae) nests. Journal of Applied Entomology 128: 204–211.

[6] Bonetto AA (1959) *Las hormigas “cortadoras” de la provincia de Santa* Fé (Géneros: *Atta* y *Acromyrmex*). Santa Fe: Ministerio de Agricultura y Ganaderia, Provincia de Santa Fe, Argentina, pp. 77.

[7] Verza SS, Forti LC, Lopes JFS, Hughes WOH (2007) Nest architecture of the leaf-cutting ant *Acromyrmex rugosus rugosus* . Insectes Sociaux 54: 303–309 10.1007/s00040-007-0943-8)

[8] Clark RM, Fewell JH (2014) Transitioning from unstable to stable colony growth in the desert leafcutter ant *Acromyrmex versicolor* . Behavioral Ecology and Sociobiology 68: 163–171 10.1007/s00265-013-1632-4)

[9] Pereira-da-Silva V, Forti LC, Cardoso LG (1981) Dinâmica populacional e caracterização dos ninhos de *Acromyrmex coronatus* (Fabricius, 1804) (Hymenoptera: Formicidae). Rev Bras Entomol 25: 87–93.

[10] Fowler HG, Forti LC, Pereira-da-Silva V, Saes NB (1986) Economics of grass-cutting ants. In: Fire ants and Leaf-cutting ants: Biology and Management. Ed. By Lofgren CS, Vander Meer RK Boulder and London: Westview Press, 18–35.

[11] Covelo De Zolessi L, Anibal Gonzalez L (1978) Observaciones sobre el género *Acromyrmex* en el Uruguay. IV. A. (*Acromyrmex*) *lundi* (Guérin, 1938) (Hymenoptera: Formicidae). Revista de la Facultad de Humanidades y Ciencias. Serie Ciencias Biológicas 1: 9–28.

[12] Jonkman JCM (1980) The external and internal structure and growth of nests of the leaf-cutting ant *Atta vollenweideri* Forel, 1983 (Hym. : Formicidae) Part I. Zeitschrift für angewandte Entomologie 89: 158–173.

[13] Lopes JFS, Ribeiro LF, Brugger MS, Camargo RS, Caldato N, et al (2011) Internal architecture and population size of *Acromyrmex subterraneus molestans* (Hymenoptera, Formicidae) nests: Comparison between a rural and an urban area. Sociobiology 58: 1–13.

[14] Bollazzi M, Forti LC, Roces F (2012) Ventilation of the giant nests of *Atta* leaf-cutting ants: does underground circulating air enter the fungus chamber? Insectes Sociaux 59: 487–498 10.1007/s00040-012-0243-9)

[15] Jacoby M (1953) Die Erforschung des Nestes der Blattschneider-Ameise *Atta sexdens rubropilosa* Forel (mittels des Ausguβverfahrens in Zement) Teil I. Zeitschrift für angewandte Entomologie 34: 145–169.

[16] Moser JC (1963) Contents and structure of *Atta texana* nest in summer. Annals of the Entomological Society of America 56: 286–291.

[17] Wetterer JK, Gruner DS, Lopez JE (1998) Foraging and nesting ecology of *Acromyrmex octospinosus* (Hymenoptera: Formicidae) in a Costa Rican tropical dry forest. Florida Entomologist 81: 61–67.

[18] Camargo RdS, Forti LC, Lopes JF, Andrade APP (2004) Characterization of *Acromyrmex subterraneus brunneus* (Hymenoptera: Formicidae) young nests in a fragment of the Neotropical forest. R Arvore, Vicosa-MG 25: 309–312.

[19] Navarro JG, Jaffe K (1985) On the adaptive value of nest features in the grass-cutting ant *Acromyrmex landolti* . Biotropica 17: 347–348.

[20] Lapointe SL, Serrano MS, Jones PG (1998) Microgeographic and vertical distribution of *Acromyrmex landolti* (Hymenoptera: Formicidae) nests in a neotropical savanna. Environmental Entomology 27: 636–641.

[21] Hölldobler B, Wilson EO (1990) The Ants. Cambridge, Massachusetts, Belknap Press, pp 596–608.

[22] Fröhle K, Roces F (2012) The determination of nest depth in founding queens of leaf-cutting ants (*Atta vollenweideri*): idiothetic and temporal control. Journal of Experimental Biology 215: 1642–1650 10.1242/jeb.066217) 22539731

[23] Diehl-Fleig E, Lucchese de Paula ME (1992) Nest foundation by *Acromyrmex striatus* (Hymenoptera, Formicidae). Biology and Evolution of Social Insects (JBillen, Ed.) Leuven University Press, Leuven (Belgium), 51–54.

[24] Franks NR, Deneubourg JL (1997) Self-organizing nest construction in ants: individual worker behavior and the nest's dynamics. Animal Behaviour 54: 779–796 10.1006/anbe.1996.0496) 9344432

[25] Rassé P, Deneubourg JL (2001) Dynamics of nest excavation and nest size regulation of *Lasius niger* (Hymenoptera: Formicidae). Journal of Insect Behavior 14: 433–449 10.1023/A:1011163804217)

[26] Buhl J, Deneubourg JL, Grimal A, Theraulaz G (2005) Self-organized digging activity in ant colonies. Behavioral Ecology & Sociobiology 58: 9–17 10.1007/s00265-004-0906-2)

[27] Jacoby M (1937) Das räumliche Wachsen des *Atta*-Nestes vom 50. bis zum 90. Tage (Hym. Formicidae). Revista de Entomologia 7: 416–425.

[28] Camargo RS, Forti LC, Fujihara RT, Roces F (2011) Digging effort in leaf-cutting ant queens (*Atta sexdens rubropilosa*) and its effects on survival and colony growth during the claustral phase. Insectes Sociaux 58: 17–22 10.1007/s00040-010-0110-5)

[29] Jacoby M (1936) Über das Wachsen des *Atta*-Nestes im ersten Jahre nach der Gründung (Hym. Formicidae). Revista de Entomologia 6: 120–126.

[30] Jacoby M (1955) Die Erforschung des Nestes der Blattschneider-Ameise *Atta sexdens rubropilosa* Forel (mittels des Ausguβverfahrens in Zement) Teil II. Zeitschrift für angewandte Entomologie 37: 129–152.

[31] Cassill D, Tschinkel WR, Vinson SB (2002) Nest complexity, group size and brood rearing in the fire ant, *Solenopsis invicta* . Insectes Sociaux 49: 158–163.

[32] Kleineidam C, Roces F (2000) Carbon dioxide concentrations and nest ventilations in nests of the leaf-cutting ant *Atta vollenweideri* . Insectes Sociaux 47: 241–248.

[33] Moser JC (2006) Complete excavation and mapping of a Texas leafcutting ant nest. Annals of the Entomological Society of America 99: 891–897 10.1603/0013-8746(2006)99891:CEAMOA2.0.CO2)

[34] Jacoby M (1960) Eine besondere Art von Hohlräumen in Nestern der *Atta sexdens rubropilosa* Forel. Zeitschrift für angewandte Entomologie 46: 34–41 10.1111/j.1439-0418.1960.tb01364.x)

[35] Quinlan RJ, Cherrett JM (1978) Aspects of the symbiosis of the leaf-cutting ant *Acromyrmex octospinosus* and its food fungus. Ecological Entomology 3: 221–230 10.1111/j.1365-2311.1978.tb00922.x)

[36] Powell RJ, Stradling DJ (1986) Factors influencing the growth of *Attamyces bromatificus*, a symbiont of Attine ants. Transactions of the British Mycological Society 87: 205–213 10.1016/S0007-1536(86)80022-5)

[37] Bollazzi M, Roces F (2002) Thermal preference for fungus culturing and brood location by workers of the thatching grass-cutting ant *Acromyrmex heyeri* . Insectes Sociaux 49: 153–157 10.1007/s00040-002-8295-x)

[38] Roces F, Kleineidam C (2000) Humidity preference for fungus culturing by workers of the leaf-cutting ant *Atta sexdens rubropilosa* . Insectes Sociaux 47: 348–350 10.1007/PL00001710)

[39] Bollazzi M, Kronenbitter J, Roces F (2008) Soil temperature, digging behavior, and the adaptive value of nest depth in South American species of *Acromyrmex* leaf-cutting ants. Oecologia 158: 165–175 10.1007/s00442-008-1113-z) 18668265

[40] Weber NA (1966) Fungus-growing ants. Science 153: 587–604. 17757227 10.1126/science.153.3736.587

[41] Bollazzi M, Roces F (2007) To build or not to build: circulating dry air organizes building responses for climate control in the leaf-cutting ant *Acromyrmex ambiguus* . Animal Behaviour 74: 1349–135.

[42] Bollazzi M, Roces F (2010) Control of nest water losses through building behavior in leaf-cutting ants (*Acromyrmex heyeri*). Insectes Sociaux 57: 267–273 10.1007/s00040-010-0081-6)

[43] Weber NA (1957) Dry season adaptations of fungus-growing ants and their fungi. The Anatomical Record 128: 638.

[44] Fröhle K (2009) Mechanismen zur Regulierung der Nestgröβe während des Koloniewachstums bei Blattschneiderameisen. PhD dissertation, Julius-Maximilians-Universität Würzburg, Germany.

[45] Fröhle K, Roces F (2009) Underground agriculture: the control of nest size in fungus-growing ants. In: *From insect nests to human architecture*- Workshop on engineering principles of innovation in swarm-made architecture (Eds. G. Theraulaz, R. Solé & P. Kuntz) European Centre for Living Technology, Venice, Italy, 95–104.

[46] Lopes JFS, Hughes WHO, Camargo RS, Forti LC (2005) Larval isolation and brood care in *Acromyrmex* leaf-cutting ants. Insectes Sociaux 52: 333–338 10.1007/s00040-005-0816-y)

[47] Armitage SAO, Fernández-Marin H, Wcislo WT, Boomsma JJ (2012) An evaluation of the possible adaptive function of fungal brood covering by Attine ants. Evolution 66: 1966–1975. 22671560 10.1111/j.1558-5646.2011.01568.x

[48] Ritter N, Cooper J (2009) New resolution independent measures of circularity. Journal of Mathematical Imaging and Vision 35: 117–127 10.1007/s10851-009-0158-x)

[49] Rudolph SG, Loudon C (1986) Load size selection by foraging leaf-cutter ants (*Atta cephalotes*). Ecological Entomology 11: 401–410.

[50] Anderson C, Jadin JLV (2001) The adaptive benefit of leaf transfer in *Atta colombica* . Insectes Sociaux 48: 404–405 10.1007/PL00001798)

[51] Deneubourg JL, Franks NR (1995) Collective control without explicit coding: The case of communal nest excavation. Journal of Insect Behavior 8: 417–432 10.1007/BF01995316)

[52] Mikheyev AS, Tschinkel WR (2004) Nest architecture of the ant *Formica pallidefulva*: structure, costs and rules of excavation. Insectes Sociaux 51: 30–36 10.1007/s00040-003-0703-3)

[53] Pielström S, Roces F (2012) Vibrational communication in the spatial organization of collective digging in the leaf-cutting ant *Atta vollenweideri* . Animal Behaviour 84: 743–752 10.1016/j.anbehav.2012.07.008)

[54] Pielström S, Roces F (2013) Sequential soil transport and its influence on the spatial organization of collective digging in leaf-cutting ants. PlosOne 8: e57040 10.1371/journal.pone.0057040) PMC3574050 23457648

[55] Toffin E, Di Paolo D, Campo A, Detrain C, Deneubourg JL (2009) Shape transition during nest digging in ants. Proceedings of the National Academy of Sciences of the United States of America 106: 18616–18620 10.1073/pnas.0902685106) 19846774 PMC2773997

[56] Toffin E, Kindekens J, Denoubourg JL (2010) Excavated substrate modulates growth instability during nest building in ants. Proceedings of the Royal Society B 277: 2617–2625 10.1098/rspb.2010.0176) 20410036 PMC2982035

[57] Minter NJ, Franks NR, Robson Brown KA (2012) Morphogenesis of an extended phenotype: four-dimensional ant nest architecture. Journal of the Royal Society Interface 9: 586–595 10.1098/rsif.2011.0377) PMC3262427 21849386

[58] Sudd JH (1969) The excavation of soil by ants. Zeitschrift für Tierpsychologie 26: 257–276.

